# Health behaviours associated with video gaming in adolescent men: a cross-sectional population-based MOPO study

**DOI:** 10.1186/s12889-020-08522-x

**Published:** 2020-03-30

**Authors:** Tuulia Puolitaival, Mirjam Sieppi, Riitta Pyky, Heidi Enwald, Raija Korpelainen, Marjukka Nurkkala

**Affiliations:** 1 Department of Sports and Exercise Medicine, Oulu Deaconess Institute Foundation sr, Albertinkatu 18A, P.O. Box 365, 90100 Oulu, Finland; 2 Center for Life Course Health Research, Faculty of Medicine, University of Oulu, P.O. Box 5000, 90014 Oulu, Finland; 3 Medical Research Center, Oulu University Hospital and University of Oulu, P.O. Box 5000, 90014 Oulu, Finland; 4 Research Unit of Medical Imaging, Physics and Technology, Faculty of Medicine, University of Oulu, P.O. Box 8000, 90014 Oulu, Finland; 5 Information Studies, Faculty of Humanities, University of Oulu, P.O. Box 8000, 90014 Oulu, Finland

**Keywords:** Video games, Physical activity, Eating habits, Male, Adolescent, Health behaviour, Gaming

## Abstract

**Background:**

Playing video games, a form of sedentary behaviour, is associated with poor well-being and increased risk of morbidity due to chronic disease. However, the association between health behaviours and video gaming is poorly understood. The purpose of this population-based study was to reveal the differences in dietary habits and physical activity for adolescent men with high amount of video games on weekdays, as compared to their peers who play less often.

**Methods:**

Seven hundred ninety-six adolescent men (age: mean = 17.8, SD = 0.6) attended compulsory conscription for military service in 2013 and completed a questionnaire regarding the amount and frequency of their video gaming. They also participated in a medical examination and underwent physiological measurements. The participants who played video games more than 3 h/d on weekdays were compared with those who played 3h/d or less. The association between health behaviours and the amount of playing video gaming was analysed using multivariable logistic regression analysis.

**Results:**

24.1% (*n* = 192) of the participants reported video gaming in excess of 3 h/d. This group had higher incidence of having low physical fitness, having poor eating habits, and being obese. No differences were found in smoking or alcohol drinking habits. Other factors, including low leisure-time physical activity (OR = 1.94; 95% CI, 1.29–2.91), low consumption of vegetables and fruits (OR = 0.83; 95% CI, 0.72–0.97), high consumption of sweetened soft drinks (OR = 1.28; 95% CI, 1.06–1.55) and high amount of sitting time (OR = 1.40; 95% CI, 1.28–1.52), explained one-fourth of the difference.

**Conclusion:**

In this population-based study, adolescent men who played video games a lot on weekdays had lower physical fitness, were more often obese, and had poorer dietary habits, as compared to their peers who played less often. Because playing video games typically adds to a person’s total sedentary time, this activity may be associated with adverse health outcomes at a very young age— especially in combination with poor health behaviours. The results of this study can be utilized to promote health interventions targeted at adolescent men so as to raise their awareness of the disadvantages of excessive video gaming.

## Background

According to the current evidence, regular physical activity in adolescence is associated with many health benefits [1]. In addition, the evidence suggests that excessive sedentary behaviour, especially sitting, is harmful to the health of young people and it may increase the risk of cardiovascular risks in adolescence [2, 3]. Sedentary behaviour has been defined as any waking behaviour that is characterized by an energy expenditure ≤1.5 metabolic equivalents (METs) and that involves a sitting, reclining, or lying posture [4]. Common sedentary behaviours include screen-time activities (e.g., watching television or other video content, playing video games, and using computers) [5], as well as driving and reading. A video game is an interactive game that is played on a digital device and that contains visual and (often) audio components; a video game can be based on a story [6].

Traditional television watching has declined in the past 10 years, and it has been replaced by other screen-based activities [7]. In their leisure time, adolescents (aged 13–18) use screen-based media, on average, 6 h 40 min per day [8]. In a previous study, 15–18 years male and female adolescents reported to play video games approximately 13.0 h/wk. [9] The other self-reported results are similar for Finnish sample with young adults (mean age 18.7 years) playing video games 11.5 h/week, and for German population-based sample, with adolescents (mean age 15.3 years) playing video games 141 min/d [10, 11]. In these all studies, boys played significantly more than girls.

However, it is worth remembering that *sedentary behaviour* is not a synonym for *physical inactivity*, which refers to physical activity levels that do not meet recommendations [12]. According to the World Health Organization’s Global Recommendations on Physical Activity for Health, children aged 5–17 should engage in at least 60 min of moderate to vigorous physical activity every day, and most of this activity should be aerobic [13]. Vigorous-intensity activities should be incorporated, including those that strengthen muscle and bone, at least 3 times per week. According to the Health Behaviour in School-aged Children study, only 17% of Finnish boys at age 15 reported at least one hour daily moderate to vigorous physical activity [14]. Instead, of 15–24 years old European men, 32% reported moderate and 27% vigorous physical activity in 4–7 days during the past week [15].

The Physical Activity Guidelines for Americans account for daily sedentary behaviour [16]. The Canadian movement guidelines for children and youth include recommendations for physical activity, sedentary behaviour, and sleep; for example, for children aged 5–17, screen time should be limited to 2 h/d [17]. The Physical Activity Guidelines for Americans also emphasize that reducing sitting time and increasing movement have enormous benefits for everyone—regardless of sex, age, ethnicity, race, or current fitness level [16].

The proportion of overweight adolescents has increased, which is problematic because adolescence weight problems often lead to adult obesity and related health problems [18, 19]. Low physical activity and high levels of sedentary behaviour are also associated with increased body mass index (BMI) and junk-food consumption [2, 20]. In addition, high screen time is associated with high consumption of energy-dense and micronutrient-poor products, as well as with low consumption of vegetables and fruits [21]. However, the association between childhood obesity and increased screen time is unclear. According to other researchers, among children aged 9–16, more time watching television and playing video games, as well as more total screen time, are associated with higher BMI for girls but not for boys [5].

The current evidence regarding the association between video gaming and health behaviours (eg, physical activity and dietary habits) is scant. In order to decrease the sedentary behaviour among youth, different patterns of sedentary behaviour should be identified and studied how those patterns are associated with physical activity and other health behaviour. Thus, this study is intended to reveal any existing differences in dietary habits and physical activity among adolescent men who play more video games than their peers do. The hypothesis of the study is that those who play more video games are less physically active and have less healthful eating habits.

## Methods

### Study design and setting

This cross-sectional study is based on a population-based study MOPO of all conscription-aged adolescent men in the years 2009–2013 in the city of Oulu, Finland. The “MOPO” is a slang word for a young recruit, and the study was named along that. In that country, conscription occurs every year and is compulsory for all male citizens in the year when they turn 18. The participants completed a medical examination before their conscription. They then filled out a questionnaire on their health, health behaviours, socioeconomic factors, and gaming habits. They also underwent physiological measurements. This study is based on the data collected during 2013 because the questionnaire included items on sedentary video gaming in that year. In 2013, 1265 adolescent men in Oulu participated in conscription, of whom 1023 (80.9%) completed the questionnaire. Thus, the data analysis for this study includes only those who reported their daily amount of video gaming (*n* = 796, 62.9%), of which 578 (72.6%) took part in the physiological measurements.

The study was conducted in accordance with the 1964 Declaration of Helsinki and was approved by the Ethical Committee of Northern Ostrobothnia Hospital District (ETTM123/2009). The participants provided written informed consent after they received a complete description of the study. The subjects had the right to refuse to participate in the study or to withdraw from it, and the study did not affect either the participants’ military service or their future healthcare.

### Questionnaire

We mostly utilized validated and widely used questions in this study. Some questions were developed for the purpose of the study. The amount of video gaming was measured with open-ended questions separately for multiplayer and single-player games: “How many hours per day, on average, do you play games on weekdays?”. Because we supposed that playing on weekdays sorts out a lot and moderately players from each other, the total hours were summed and divided into tertiles. The highest tertile was defined as playing > 3 h/d on weekdays and labelled *PV > 3*, and the other two tertiles were combined (thus defined as playing ≤ 3 h/d on weekdays) and labelled *PV ≤ 3*. Video game playing on weekends and days off was determined through similar questions, which was used in the characteristics (Table 1).

**Table 1:** Characteristics of the population-based sample of adolescent men based on amount of video gaming on weekdays

Characteristics	n	All (***n*** = 796)	Video Gaming > 3 h/day (***n*** = 192)	Video Gaming ≤ 3 h/day (***n*** = 604)	***P*** value^a^
Age^b^, y	792	17.9 (0.7)	18.1 (0.9)	17.9 (0.7)	.008
Weight, kg	577	73.0 (14.1)	75.4 (17.8)	72.2 (12.6)	.102
Height, cm	578	178.1 (6.3)	178.2 (7.0)	178.2 (6.1)	.718
**BMI**^c^, kg/m^2^	577	23.0 (4.3)	23.8 (5.5)	22.7 (3.8)	.107
Underweight (BMI < 18.5), No. (%)		47 (8.1)	10 (7.5)	37 (8.4)	.858
Normal weight (18.5–24.9) No. (%)		397 (68.8)	88 (65.7)	309 (69.8)	.395
Overweight (BMI 25–29.9) No. (%)		96 (16.6)	20 (14.9)	76 (17.2)	.598
Obesity (BMI ≥ 30), No. (%)		37 (6.4)	16 (11.9)	21 (4.7)	.008
**Body fat**, %	576	16.5 (8.4)	19.3 (10.0)	15.6 (7.6)	<.001
**Waist circumference**, cm	577	82.0 (10.4)	84.7 (13.2)	81.1 (9.1)	.011
Muscle mass, %	576	47.0 (4.8)	45.3 (5.6)	47.6 (4.4)	<.001
Self-rated health	776				.001
Poor, No. (%)		56 (7.2)	24 (12.8)	32 (5.4)	
Good or average, No. (%)		720 (92.8)	164 (87.2)	556 (94.6)	
**Grip strength,** kg	576	45.0 (7.5)	43.8 (7.8)	45.4 (7.4)	.017
Light and brisk physical activity, median (interquartile range), MET-min weekly	769	1013 (413–1875)	615 (204–1286)	1148 (563–2085)	<.001
**< 500 MET-min**, No. (%)		219 (28.5)	88 (47.3)	131 (22.5)	<.001
≥ 500 MET-min, No. (%)		550 (71.5)	98 (52.7)	452 (77.5)	
**Aerobic fitness (Polar Fitness Test)**, mL/min/kg	563	52.9 (7.1)	50.4 (6.6)	53.7 (7.1)	<.001
Poor self-rated physical fitness, No. (%)	781	190 (24.3)	84 (44.4)	106 (17.9)	<.001
**Sitting during leisure time**, h/d	762	4.2 (2.3)	5.8 (2.4)	3.7 (2.0)	<.001
≥ 5 h/d, No. (%)		273 (35.8)	129 (69.0)	144 (25.0)	<.001
< 5 h/d, No. (%)		489 (64.2)	58 (31.0)	431 (75.0)	
Mean consumption frequency, d/wk					
**Vegetables and fruits**	744	2.0 (1.5)	1.6 (1.5)	2.2 (1.5)	<.001
**Sweetened soft drinks and energy drinks**	750	1.1 (1.0)	1.4 (1.2)	1.0 (0.9)	<.001
Salty foods	747	1.0 (0.9)	1.1 (1.0)	1.0 (0.8)	.499
Sweet foods	752	1.5 (1.1)	1.5 (1.2)	1.5 (1.0)	.455
Usually eats breakfast	786				<.001
Yes, No. (%)		575 (73.2)	118 (62.8)	457 (76.4)	
No, No. (%)		211 (26.8)	70 (37.2)	141 (23.6)	
Smokes currently, No. (%)	784				.851
Yes, No. (%)		209 (26.7)	52 (27.4)	157 (26.4)	
No, No. (%)		575 (73.2)	128 (72.6)	437 (73.6)	
Uses snuff currently, No. (%)	787	189 (24.0)	144 (23.4)	145 (24.2)	.846
Binge drinks alcohol ≥1/wk., No. (%)	786	172 (21.9)	43 (23.1)	129 (21.5)	.685

^a^ This is the *P* value between groups for video gaming > 3 h/day vs. video gaming ≤ 3 h/day

^b^ Values are expressed as mean (SD) unless otherwise stated. Some numbers do not match due to missing values

^c^ Variables in bold are part of the binary logistic regression

Abbreviation: *BMI* body mass index

The amount of leisure-time physical activity was measured with the following question: “How often, and for how long, do you participate in light or brisk physical activity or exercise during your leisure time?” *Brisk physical activity* was defined as causing at least some sweating and heavy breathing, and *light physical activity* was defined as causing no sweating or heavy breathing. This question had 6 response options for frequency (*once per month or less* [score 0 in calculation], *2–3 times per month* [0.5], *once per week* [1], *2–3 times per week* [2.5], *4–6 times per week* [5], and *daily* [7]), as well as 6 options for duration (*not at all* [0 min], *less than 20 min* [10], *20–39 min* [30], *40–59 min* [45], *60–90 min* [75], and *> 90 min* [90]) [22]. Weekly averages in MET minutes for light or brisk physical activity were calculated by multiplying the physical activity’s volume (duration [min] × frequency) by its intensity (light physical activity = 3 METs and brisk physical activity = 5 METs) [23]. The resulting values were then summed, and a dichotomous variable with a cut-off point of 500 MET min was created from the aggregate MET min values (from the Physical Activity Guidelines for Americans) [24].

The amount of leisure time spent sitting each day was determined with the following question: “Approximately how much time do you spend sitting per day, outside of school and work (for example, watching television, reading, using a computer, playing video games, or using the Internet)?” The participants reported this in hours, and the results were classified as a two-class variable (nonsedentary vs. sedentary) with a cut-off point of 5 h/d [25], and sitting hours were used as a continuous variable in the logistic regression analysis.

Physical fitness was evaluated with the question “How fit are you, as compared to your peers?” The response options were on a 5-point scale from 1 (*significantly poorer*) through 5 (*significantly better*). The values of 1 and 2 were combined as *poorer*, and the values of 3–5 were combined as *similar or better*, resulting in a two-class variable. Similarly, *self-rated health* was investigated with the following question: “What is the state of your health?” The response options were on a 5-point scale from 1 (*good*) through 5 (*poor*). Again, the values from 1 to 3 were combined (*good or average*), as were the values of 4 and 5 (*poor*).

Dietary habits were determined with the following question about the mean consumption of various foods: “How often during the preceding week did you consume the following foods or drinks?” The answers were 1 (*never*), 2 (*1–2 days per week*), 3 (*3–5 days per week*), and 4 (*6–7 days per week*), and the frequency was recoded using the following conversion: *never* = 0 d/wk., *1–2 days per week* = 1.5 d/wk., *3–5 days per week* = 4 d/wk., and *6–7 days per week* = 6.5 d/wk. [26, 27] The mean frequency of consuming fresh vegetables, cooked vegetables, and fruits was combined into one variable by summing the recoded frequencies for each and then dividing by three. The mean consumption of junk food was similarly the average of the frequencies for salty foods and sweet foods. *Salty foods* were defined as including “pizza and kebabs,” “savoury pastries (burgers, pies, etc.),” and “salty snacks (crisps, etc.).” *Sweet foods* were defined as including “sweet pastries (buns, biscuits, etc.)” and “chocolate and other sweets.” The mean consumption of sweetened soft drinks was based on the average of the frequencies for sugary or artificially sweetened soft drinks and energy drinks. The participants also answered whether they usually eat breakfast (yes or no).

Alcohol intake was measured with this question: “How often do you drink six or more servings of alcohol at once?” The response options were on a 6-point scale from 1 (*never*) through 6 (*daily or almost daily*). A two-grade variable was formed to measure binge drinking (defined as 6 servings or more in a day at least once per week) [25, 28]. The participants also reported whether they currently smoked or used snuff (yes or no for each).

### Physiological measurements

Each participant’s height was measured (with 0.5-cm precision) using a wall-mounted measuring tape*.* Similarly, waist circumference was measured (to the nearest 1 cm) along the midline between the lowest rib and the iliac crest. Body composition was measured using weight (with 0.1-kg accuracy), fat-free mass (at 0.1-kg accuracy), and percentage body fat; the latter was measured with direct segmental multifrequency bioelectric impedance analysis (InBody720, Biospace Co., Ltd., Seoul, Korea). During the measurements, the subjects stood without shoes or socks and in light indoor clothing. This method has been validated using whole-body dual X-ray absorptiometry. Each participant’s percentage of fat-free mass was then calculated by dividing muscle mass (kg) by whole-body mass (kg), and each participant’s BMI was calculated by dividing weight (kg) by height squared (m^2^).

Each participant’s grip strength was measured using a hand dynamometer (Saehan Corporation, Korea). During the test, each subject stood with legs apart and elbows at a 90° angle, gripping the dynamometer with maximum force. After two attempts using each hand, the best result for each hand was recorded, and the mean of these results was used in the analyses. Aerobic fitness was evaluated using the Polar Fitness Test (Polar Electro, Finland), which was conducted while the subject was at rest for 5 min. This test predicts maximal oxygen uptake (mL/min/kg) using resting heart rate, heart-rate variability, sex, age, height, body weight, and self-assessed physical activity [29]. The Polar Fitness Test high accuracy (mean error: 6.5%) and correlates highly with the ergo-spirometry test as a measure of aerobic condition (0.96) [30].

### Statistical analysis

Statistical analyses were performed using IBM SPSS Statistics for Windows (Version 24.0, Armonk, New York, IBM Corp). An independent-sample *t* test for continuous variables was used to analyse the statistical significance of the mean differences with 95% confidence intervals between the PV > 3 and PV ≤ 3 groups. Due to the skewed distributions of MET min values, the medians of these values were used, as were the results of a Mann-Whitney *U* test. Cross-tabulation and the chi squared test were used for the categorical variables. All variables that were significantly associated with video gaming in the univariate analysis were then entered into the binary logistic regression analysis. Video game playing was a dependent variable with categories: 0 = *playing ≤ 3 h/d on weekdays* and 1 = *playing > 3 h/d on weekdays*. Because playing video games is a type of sedentary behaviour, leisure-time sitting (as a continuous variable) was entered into the block one with enter selection, and the other associated variables in the second block with forward stepwise (Likelihood Ratio) selection. The level of significance was set at *P* < .05.

## Results

The study population comprised 796 adolescent men with mean age 17.9 (age range 17–22). The PV > 3 group included 192 (24.1%) individuals, who played video games, on average, 5.5 (SD = 2.0) h/d on weekdays and 7.3 (SD 3.9) h/d on weekends and days off. The PV ≤ 3 group included 604 individuals, who played, on average, 1.3 (SD 1.1) h/d on weekdays and 2.4 (SD 2.2) h/d on weekends and days off. The difference on playing on weekends and days off was statistically significant between the groups (mean difference 4.9 (95% CI 4.3–5.5).

The characteristics of the study population are described in Table 1. The PV > 3 group included more overweight individuals than the PV ≤ 3 group (*P* = .007), but there was no difference in the number of underweight individuals. In addition, those in the PV > 3 group had, on average, 3.4% higher body fat (95% CI, 1.5–5.3%; *P* < .001), 3.1 cm larger waist circumference (95% CI, 0.7–5.4 cm; *P* = .011), and 2.1% less muscle mass (95% CI, 1.0–3.1%; *P* < .001), as compared to those in the PV ≤ 3 group. The participants in the PV > 3 group had poorer physical fitness, on average: 1.8 kg weaker grip strength (95% CI, 0.3–3.2 kg; *P* = .017) and 3.2 mL/min/kg higher maximal oxygen uptake (95% CI, 1.9–4.6 mL/min/kg; *P* < .001). They also had less physical activity, as measured in MET min (median = 88 in the PV > 3 group vs. median = 131 in the PV ≤ 3 group, *P* < .001). In the PV > 3 group, 44% rated their physical fitness as poorer than that of their peers, but in the PV ≤ 3 group, only 18% did so (*P* < .001). In the PV > 3 group, 12.8% rated their health as poorer than that of their peers, but in the PV ≤ 3 group, only 5.4% did so (*P* = .001). Those who played a lot of video games also sat a lot; in the PV > 3 group, 69% of the participants sat for 5 h/d or more during their leisure time, but in the PV ≤ 3 group, only 25% did so (*P* < .001). Eating habits were also poorer in the PV > 3 group; during the preceding week, those in this group ate vegetables and fruits less frequently (mean difference 0.5; 95% CI, 0.3–0.8 times per week; *P* < .001) and drank sweetened soft drinks and energy drinks more often (mean difference 0.4; 95% CI, 0.2–0.6 times per week; *P* < .001). In addition, those in the PV > 3 group skipped breakfast more often than did those in the PV ≤ 3 group (*P* < .001).

In the binary logistic regression analysis (Table 2), playing more video games on weekdays was significantly associated with all of the following: lower mean frequency of consuming vegetables and fruits (OR = 0.83; 95% CI, 0.72–0.97), higher mean frequency of consuming sweetened soft drinks and energy drinks (OR = 1.28; 95% CI, 1.06–1.55), higher leisure-time sitting (OR = 1.40; 95% CI, 1.28–1.52) and less leisure-time physical activity (OR = 1.94; 95% CI, 1.29–2.91). This model explained 25.1% of the OR for high levels of video gaming.

**Table 2:** Factors associated with a high amount of video gaming on weekdays (> 3 h/d) in the adolescent men (*n* = 698) in the logistic regression analysis^a^

Variables	Adjusted OR	95% CI	*P* Value
Block 1: method = enter			
Sitting during leisure time, hours	1.40	1.28–1.52	<.001
Block 2: method = forward stepwise (LR)			
Low leisure-time physical activity^b^ (vs. ≥ 500 MET-min weekly)	1.94	1.29–2.91	.001
Mean consumption frequency of vegetables and fruits, d/wk	0.83	0.72–0.97	.014
Mean consumption frequency of sweetened soft drinks and energy drinks, d/wk	1.28	1.06–1.55	.010

*Note*: Variables in bold in the Table 1 were significantly associated (*p* < .05) with high amount of video gaming (ref. group: ≤ 3 h/d playing video games) in univariate analysis and were entered in the binary logistic regression analysis. This is the final model resulting of the 3rd step

^a^ Nagelkerke *R*^2^ = 25.1%

^b^ Total light and brisk MET < 500 min

## Discussion

This population-based cross-sectional study of adolescent men mainly 17–18 years old related to the associations that both physical activity and dietary habits have with the amount of video gaming on weekdays. Having low physical fitness, being obese, and having poor eating habits were more frequent among those who played video games more than 3 h/d on weekdays. No difference was found in smoking and alcohol drinking. Low leisure-time physical activity, low frequency of consumption of vegetables and fruits, high consumption of sweetened soft drinks and energy drinks, and high amount of leisure sitting time explained one-fourth of the results.

High screen time is also associated with insufficient physical activity and higher sitting time, according to studies of 13- to 18-year-old boys and girls; in these studies, screen time included watching television, using a computer, playing video games, and using smartphones [31, 32]. Video game playing has been negatively associated with physical activity among undergraduate men, especially among those who play online video games [33]. In addition, in a Spanish school-based study the adolescent boys who reported having weekly at least 4 h screen time more unlikely met the recommendation for moderate to vigorous physical activity [34]. This study’s results indicate that there is a connection between high levels of video gaming and physical inactivity and sedentary behaviour. Researchers have reported that screen time is positively related to BMI in those aged 11–19 [33, 35, 36]. In addition, Ballard et al. [33] found a correlation between playing video games and high BMI. Although this study’s results indicate that there is no difference in weight and BMI between the groups, the PV > 3 group included more obese (BMI ≥ 30) adolescent men than the PV ≤ 3 group. This study’s association between a high amount of video gaming and poor self-rated health also supports the previous findings [37, 38].

Poor eating habits are also associated with playing video games. The results of a previous study of fourth-grade children show a positive association between video gaming and consumption of high-calorie and low-nutrient foods [39]. Another study’s results indicate that 2 h/d or more of screen time is associated with increased energy intake [40], such that adolescents with more screen time consume more sugar-sweetened beverages [32, 41]. Researchers have also suggested that those with high screen time tend to consume fewer vegetables and fruits [31, 32, 40], and our study results indicate a similar association between high video gaming and lower consumption of vegetables and fruits.

This study’s results indicate no association between alcohol consumption and the amount of video gaming, which supports the previous findings [42–44]. The previous findings on the association between smoking and video gaming are contradictory, with some researchers finding no relationship between smoking and video gaming [42, 44], but others finding the opposite [45].

The strengths of this research are its population-based design, which contained an entire age cohort of adolescent men at approximately 18 years old; its substantial data collection (questionnaire responses, measurements, and examinations); and its high compliance rate due to the data being collected during compulsory conscription. In addition, the measurements of body composition and physical performance were taken under controlled conditions. Those who did not complete the study questionnaire did not differ from the study participants with regards to perceived weight, self-rated health, sedentary behaviour, or anthropometric measures. However, those who did not complete the questionnaire were less likely to be students (76% vs. 86%, *P* < .001) and had lower self-rated fitness (29% vs. 38%, *P* = .001), as compared to their peers.

One limitation of this study is the lack of objective measurements of physical activity and time spent playing video games. In this study, we used self-reported measures, which may have led to overestimates of the participants’ amount of overall physical activity [46, 47] or of the intensity of that activity [48]. We did not reveal the real amount of consumed vegetables and fruits (as well as other studied foods) with the used variable examining the average frequency of days when consumed vegetables and fruits, for example a study participant could eat fresh vegetables every day, but never cooked vegetables or fruits, thus having a mean frequency of 2.17. The mean frequency was still used to reveal the overall consumption of vegetables and fruits, as it reflects the direction of consumed vegetables and fruits. The participants may have also encountered difficulties in recalling their food intake of last week. The study population consisted of adolescent men of specific age, which may limit the generalizability of the study results. Finally, this study was cross-sectional, so no conclusion regarding causality could be drawn.

## Conclusion

In this population-based study of approximately 18 years old men, those who played video games more than 3 hours per day on weekdays, as compared to their peers, had lower levels of physical activity and were more likely to be obese. They also consumed less vegetables and fruits, and more sweetened soft drinks. Video gaming typically increases daily sitting time, as was also in this study, which affect harmful for health with other poor health behaviours. These preliminary results of this study fortify that video gaming is associated with detrimental health behaviour, which would be worth to take account with adolescent men, who play a lot on weekdays. Because physically less active 17–18 years old men seemed to spend more time playing video games, it could be studied in the future whether video games could be used in physical activation of adolescent men. Prospective studies are also needed in order to investigate the causality of the correlation between video gaming and health behaviours in adolescent men.

## Data Availability

Data is available from the University of Oulu, for research collaborators who meet the criteria for accessing confidential data. Please, contact project center for further information: projectcenter@oulu.fi.

## Abbreviations

MET: Metabolic equivalent
BMI: Body mass index
PV > 3: Playing videogames > 3 h/d on weekdays
PV ≤ 3: Playing videogames ≤3 h/d on weekdays
